# Statin-Associated Severe Rhabdomyolysis With Mixed Neuromuscular Involvement Mimicking Guillain‑Barré Syndrome: A Case Report

**DOI:** 10.7759/cureus.107939

**Published:** 2026-04-29

**Authors:** Abhijit Chatterjee, Subhayan Bhattacharya, Jayanta Datta

**Affiliations:** 1 Internal Medicine, Charnock Hospital, Kolkata, IND; 2 Tropical Medicine, Charnock Hospital, Kolkata, IND

**Keywords:** immune-mediated necrotising myopathy, neuromuscular involvement, polyneuropathy, rhabdomyolysis, statin-associated myopathy

## Abstract

Statin-associated myopathy represents a well-recognised adverse effect of 3-hydroxy-3-methylglutaryl-coenzyme A (HMG-CoA) reductase inhibitors, ranging from myalgia to life-threatening rhabdomyolysis. Neurological involvement in the form of peripheral neuropathy mimicking Guillain-Barré syndrome (GBS) is rare and diagnostically challenging. We report a case of a 72-year-old man with diabetes mellitus, hypertension, and single-vessel coronary artery disease who underwent percutaneous transluminal coronary angioplasty (PTCA) and was initiated on atorvastatin 40 mg once daily as secondary prevention. Within two months of initiation, he developed myoglobinuria followed by rapidly evolving bilateral upper and lower limb weakness on admission. Peak creatine kinase (CK) was 48,000 U/L, with urine myoglobin exceeding 12,000 ng/mL. Nerve conduction studies demonstrated a lower-motor-neuron-type demyelinating sensorimotor polyneuropathy, while electromyography revealed a myopathic pattern. Although GBS was a primary diagnostic concern, cerebrospinal fluid analysis did not corroborate due to the absence of albuminocytologic dissociation. The patient was managed with statin discontinuation and aggressive intravenous fluid resuscitation. Myoglobinuria started to resolve by day two. At discharge, upper limb weakness had fully resolved, and lower limb power improved from 1/5 to 3/5, with CK declining to 8060 U/L. This case highlights that severe statin-associated rhabdomyolysis with mixed electrophysiological features of demyelinating polyneuropathy and myopathy can closely mimic GBS. The significant clinical improvement following statin withdrawal without immunomodulatory therapy favours a toxic neuromyopathic process over an immune-mediated aetiology, emphasising early recognition and prompt discontinuation of the offending agent to prevent complications and improve outcomes.

## Introduction

Statins are widely prescribed lipid-lowering agents that form the cornerstone of cardiovascular risk reduction in both primary and secondary prevention. 3-Hydroxy-3-methylglutaryl-coenzyme A (HMG CoA) reductase inhibitors are generally well tolerated. Although mild muscle-related adverse effects such as myalgia are relatively common, severe complications, including rhabdomyolysis with acute kidney injury and neuromuscular involvement, are rare, occurring approximately 1 per 10000 patient-years of treatment [1]. Apart from direct muscle toxicity, statins have also been implicated in immune-mediated necrotising myopathy associated with anti-HMGCR (3-hydroxy-3-methylglutaryl-coenzyme A reductase) antibodies, which is recognised as a distinct entity requiring immunomodulators within the spectrum of statin-related muscle disorders [2]. Neurological manifestations, particularly peripheral neuropathy, have only rarely been described in the literature but can considerably complicate both the diagnosis and management. This becomes especially relevant when the clinical picture resembles Guillain-Barré syndrome (GBS), which is an acute immune-mediated polyradiculoneuropathy that requires an entirely different therapeutic approach.

The coexistence of electrophysiologically confirmed myopathy, and demyelinating polyneuropathy in the same patient represents a particularly rare and poorly characterised mixed neuromuscular phenotype. Differentiating this entity from GBS is clinically important, as misdiagnosis may result in unnecessary and potentially harmful interventions like plasmapheresis or intravenous immunoglobulin (IVIG).

We report a case of severe statin-associated rhabdomyolysis with a GBS-like presentation with mixed neuromuscular involvement and systemic complications, highlighting the importance of early recognition, prompt statin withdrawal, and aggressive supportive management. This report is presented in compliance with the CARE (CAse REport) 2013 guidelines.

## Case presentation

Patient information

A 72-year-old man presented with a five-day history of reddish-brown urine, followed by progressively worsening bilateral lower limb weakness that rendered him bedbound, along with bilateral upper limb weakness, described as an inability to lift his hands above the head for three days. His medical history was notable for hypertension, type 2 diabetes mellitus, and single-vessel coronary artery disease. He had undergone PTCA two months earlier. He was initiated on atorvastatin 40 mg once daily at bedtime along with ticagrelor 90 mg twice daily and aspirin 75 mg once daily for his post-procedural secondary prevention regimen. No other potential myotoxic medications, including antifungal, fibrates, and macrolide antibiotics, were being coadministered. There was no preceding febrile illness, upper respiratory tract infection, or gastrointestinal illness in recent weeks.

Clinical findings

At presentation, the patient was conscious, alert, and oriented with stable vitals. Neurological examination showed symmetrical proximal muscle weakness in all four limbs, predominantly involving the lower limbs, with lower limb power of 1/5 and upper limb power of 3/5. Plantar responses were equivocal on both sides. There was bilateral pitting oedema. Bulbar functions were intact with no dysphasia, dysarthria, or respiratory compromise. Sensory examination demonstrated diminished sensation in glove-and-stocking distribution. Deep tendon reflexes were globally diminished. There were no cerebellar signs, and cranial nerve examination was unremarkable. There was mild tenderness on palpation in the right hypochondrium region. Urine appeared dark red-brown in colour, consistent with probable myoglobinuria. The remaining systemic examinations revealed no abnormalities.

Timeline

Table 1 summarises the clinical timeline from statin initiation to hospital discharge.

**Table 1:** Clinical timeline CK: Creatine Kinase; PTCA: Percutaneous transluminal coronary angioplasty; SVCAD: Single-vessel coronary artery disease; NCV: Nerve conduction velocity; EMG: Electromyography; IVIG: Intravenous immunoglobulin; CSF: Cerebrospinal fluid; IV: Intravenous.

Time Point	Clinical Events
Pre-admission	
~2 months before admission	Percutaneous transluminal coronary angioplasty (PTCA) was performed for single-vessel coronary artery disease (SVCAD). Atorvastatin 40 mg once daily was initiated as secondary prevention.
5 days before admission	The onset of reddish-brown discolouration of urine (myoglobinuria) was first noted by the patient.
3 days before admission	Progressive limb weakness and generalised myalgia commenced.
Hospital admission	
Day 1: Admission	The patient presented with lower limb weakness with power 1/5, proximal upper limb weakness with power 3/5, and dark red-brown urine. Investigations: Serum CK 48,000 U/L, urine myoglobin >12,000 ng/mL, and serum vitamin D 16 ng/mL (deficient). Full laboratory workup performed. Electrophysiology: NCV showed lower motor neuron-type demyelinating sensorimotor polyneuropathy; EMG showed myopathic pattern and mixed neuromuscular phenotype. Management: Atorvastatin discontinued immediately. Rigorous IV fluid resuscitation started. Vitamin D supplementation initiated. IVIG was considered.
Day 2	Myoglobinuria started to resolve, urine colour became pale red. No acute kidney injury throughout admission. CK: 32,000 U/L. CSF study was normal with no albuminocytological dissociation.
Day 4	CK declining to 12,703 U/L. Urine colour normalised. Clinical improvement noted.
Days 5-7	Progressive clinical improvement. Upper limb strength returned, and lower limb power started to improve. Regular physiotherapy continued.
Day 8: Discharge	Upper limb weakness fully resolved (power 5/5). Lower limb power improved from 1/5 to 3/5. CK: 8,060 U/L. Patient discharged with physiotherapy referral and outpatient follow-up instructions.
Post-discharge	
Following discharge	The patient was lost to follow-up. Final neurological outcome, CK normalisation, and need for immunotherapy could not be ascertained.

Diagnostic assessment

Laboratory Investigations

Haematological and biochemical findings are summarised in Table 2. The peak serum CK level was 48,000 U/L (reference range: 30-200 U/L). Urine myoglobin was markedly raised at >12,000 ng/mL, consistent with severe rhabdomyolysis and associated myoglobinuria. Interestingly, despite the marked muscle injury, renal parameters remained within normal limits, with serum creatinine showing no evidence of acute kidney injury. This may have been related to an early initiation of aggressive intravenous hydration. Liver enzymes were elevated (AST > ALT), with reduced serum albumin and a borderline high international normalised ratio (INR). As tests were negative for any hepatic infection and ultrasonography (USG) of the whole abdomen was normal, this liver disjunction was likely attributed to statin usage. The patient was also found to have a vitamin D deficiency, with a 25-hydroxy vitamin D level of 16 ng/mL.

**Table 2:** Haematological and biochemical investigation findings PCV: Packed cell volume; INR: International normalised ratio; SGOT/AST: Serum glutamic oxaloacetic transaminase/aspartate aminotransferase; SGPT/ALT: Serum glutamic pyruvic transaminase/alanine aminotransferase; HAV: Hepatitis A virus; HEV: Hepatitis E virus; HMGCR: 3-hydroxy-3-methylglutaryl-coenzyme A reductase; CSF: Cerebrospinal fluid.

Category/Parameter	Result	Reference Range
Haematology		
Haemoglobin	12.4 g/dL	13.0–17.0 g/dL
PCV (Haematocrit)	37%	40%–52%
Total leucocyte count	16,100 cells/mm³	4,000–11,000 cells/mm³
Neutrophils	88%	40%–70%
INR	1.22	0.8–1.2
Inflammatory markers		
C-reactive protein (CRP)	8.57 mg/L (Elevated)	<5.0 mg/L
Lactate dehydrogenase (LDH)	3,700 U/L (Elevated)	140–280 U/L
Electrolytes and renal function		
Serum sodium	131 mEq/L (Mildly low)	135–145 mEq/L
Serum potassium	5.1 mEq/L	3.5–5.0 mEq/L
Blood urea	38 mg/dL	10–40 mg/dL
Serum creatinine	0.9 mg/dL	0.7–1.3 mg/dL
Liver function tests		
Total serum bilirubin	1.0 mg/dL	0.2–1.2 mg/dL
Direct bilirubin	0.3 mg/dL	0.0–0.3 mg/dL
Indirect bilirubin	0.7 mg/dL	0.1–1.0 mg/dL
Serum albumin	2.8 g/dL (Low)	3.5–5.0 g/dL
SGOT/AST	2,499 U/L (Markedly elevated)	10–40 U/L
SGPT/ALT	1,122 U/L (Elevated)	7–56 U/L
Muscle markers		
Serum creatine kinase (CK)	48,000 U/L (Severely elevated)	30–200 U/L
Urinalysis		
Urine colour	Dark red-brown (Myoglobinuria)	Clear/Yellow
Urine myoglobin	>12,000 ng/mL	<5 ng/mL
Metabolic parameters		
HbA1c	7.0%	<5.7% (Normal)
Serum 25-OH vitamin D	16 ng/mL (Deficient)	>30 ng/mL (Sufficient)
Infectious serology		
Anti-HAV IgM	Negative	Negative
Anti-HEV IgM	Negative	Negative
Scrub typhus IgM	Negative	Negative
Special investigations		
Anti-HMGCR antibody	Not performed (Resource constraints)	Negative
CSF analysis	No albuminocytological dissociation	—
MRI thigh	Contraindicated (Metallic implant)	—
Muscle biopsy	Refused by the patient	

Electrophysiological Study

Nerve conduction velocity (NCV) study demonstrated demyelinating sensorimotor polyneuropathy with lower motor neuron features in all four limbs. Electromyography (EMG) showed features of myopathy characterised by short-duration, low-amplitude, polyphasic motor unit action potentials with early recruitment. This combination of demyelinating polyneuropathy on NCV studies and myopathy on EMG in the same patient constitutes the mixed neuromuscular phenotype central to the report.

Other Investigations

Magnetic resonance imaging (MRI) of the bilateral thighs was planned but could not be performed because the patient had a preexisting MR-incompatible implant used for patellar fixation. Cerebrospinal fluid (CSF) analysis did not show albuminocytological dissociation, which reduced the likelihood of GBS. Anti-HMGCR antibody testing could not be undertaken because of resource limitations. Although a muscle biopsy was advised, the patient declined.

Differential Diagnosis

The primary diagnostic dilemma was between statin-associated neuromuscular toxicity and GBS. Key features favouring statin toxicity included:

1. A clear temporal relationship with atorvastatin within two months of initiation.

2. Markedly elevated CK levels, which are inconsistent with GBS, where CK is typically normal or mildly elevated.

3. A myopathic EMG pattern, which is not expected in GBS.

4. Absence of preceding infection or other GBS trigger.

5. Significant myoglobinuria, indicating severe rhabdomyolysis.

6. Rapid and sustained clinical improvement following statin discontinuation alone.

Immune-mediated necrotising myopathy (IMNM) with anti-HMGCR antibodies could not be formally excluded given resource limitations, but favourable response to statin withdrawal without immunosuppression makes a predominantly toxic rather than immune-mediated aetiology more likely in this case.

Therapeutic Intervention

Atorvastatin was discontinued immediately on admission. Aggressive intravenous fluid resuscitation was initiated with normal saline as a primary intervention strategy with a target urine output of 80-100 mL/hour to promote urinary myoglobin clearance, prevent renal tubular injury, correct dyselectrolytaemia, and maintain urine output. Myoglobinuria started resolving by day two and was completely cleared by day four, indicating effective renal protection with this approach.

Vitamin D supplementation was initiated with oral cholecalciferol 1500 mcg once a week for six weeks, following documentation of deficiency. Vitamin D deficiency is a recognised contributor to muscle weakness and myopathy. Intravenous immunoglobulin (IVIG) was considered for treatment. However, the non-contributory CSF results and clinical improvement following statin withdrawal did not require IVIG therapy. Corticosteroid therapy was initially considered in view of possible IMNM but was withheld, given the favourable clinical response to supportive measures alone and the inability to confirm anti-HMGCR positivity. Physiotherapy was continued during the hospital stay and was advised on discharge.

Follow-Up and Outcomes

**Figure 1:**
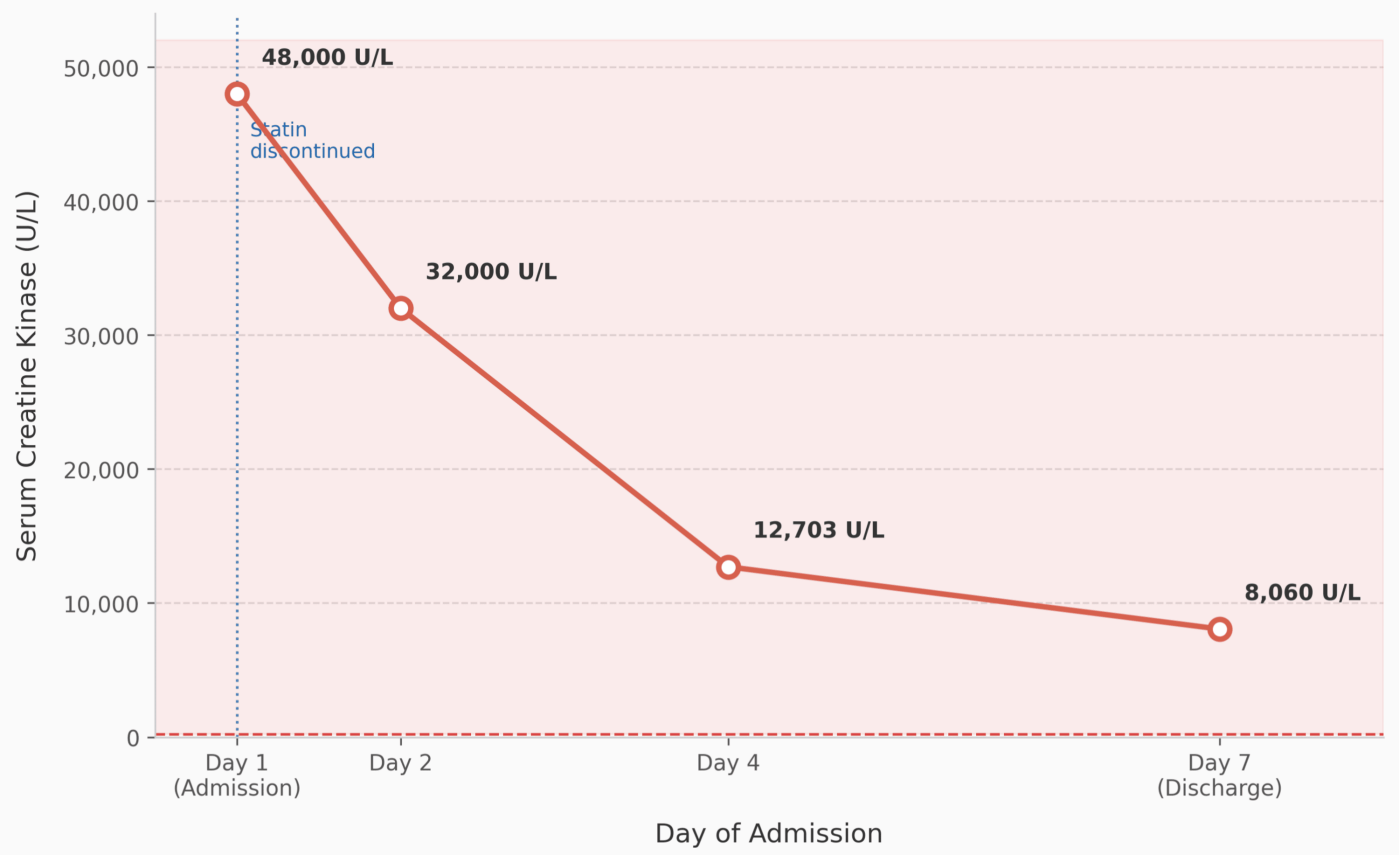
Serial serum creatine kinase (CK) trend during hospitalisation CK: Creatine kinase; U/L: Units per litre.

The patient demonstrated significant clinical improvement over the eight-day admission. Upper limb weakness resolved completely. Lower limb power improved from 1/5 on admission to 3/5 at discharge. Serum CK declined from a peak of 48000 U/L to 8060 U/L by day 7, indicating ongoing but incomplete resolution of muscle injury. Figure 1 demonstrates the trend of CK during hospital stay.

Renal function remained preserved throughout. Liver enzyme levels started to decrease. The patient was discharged with physiotherapy and single antiplatelet therapy (aspirin 75 mg once daily) as per cardiologist advice, along with follow-up instructions, including repeat CK, neurological review, and repeat NCV study. Unfortunately, the patient was lost to follow-up, and the long-term neurological outcome, final CK normalisation, and any need for subsequent immunotherapy could not be ascertained. This represents a significant limitation of this report.

## Discussion

This case illustrates several clinically important and underrecognised aspects of statin-associated neuromuscular toxicity. The simultaneous polyneuropathy on NCV studies and a myopathic pattern on EMG in the same patient, constituting a mixed neuromuscular phenotype, is the defining and most distinctive feature of this report. This electrophysiological combination is exceptionally rare in the literature on statin-associated muscle injury. The most instructive precedent is the case series reported by Collidge et al. [3], in which four patients with type 2 diabetes mellitus on long-term statin therapy developed severe rhabdomyolysis with painless flaccid areflexic paralysis of both lower limbs, following the addition of fusidic acid, which is a known pharmacokinetic interaction. In those cases, NCV reports were interpreted as GBS, resulting in delayed recognition of rhabdomyolysis and unnecessary administration of IVIG, a clinically important cautionary precedent. Another case [4] has documented statin-associated peripheral neuropathy in the absence of rhabdomyolysis with improvement following drug withdrawal. Population-level evidence was found from a study [5] that demonstrated a 4- to 14-fold increased risk of polyneuropathy among long-term statin users compared to controls, with duration and dose identified as key risk modifiers.

The clinical presentation mimicking GBS was the initial diagnostic challenge. Both conditions can present with rapid-onset limb weakness and areflexia, most commonly involving the lower limbs in GBS. However, several features in this case were atypical for GBS and characteristic of statin toxicity, such as the markedly elevated CK level of 48000 U/L (CK level is generally normal or only mildly elevated in GBS), the presence of myopathic EMG changes (not commonly expected in GBS), significant myoglobinuria, and the absence of any prodromal illness. Importantly, the dramatic clinical improvement after statin withdrawal alone without any IVIG or plasmapheresis provides compelling retrospective evidence of a statin-mediated aetiology.

The short latency of onset, approximately two months after atorvastatin initiation post-PTCA, is clinically significant. While statin-associated myopathy can occur at any point during treatment, onset within the first two months following a major cardiac procedure may reflect a combination of factors, including a post-procedure inflammatory state, pre-procedure concurrent medications, or a rare possibility of genetic susceptibility involving SLCO1B1 or CYP450 polymorphisms (though pharmacogenomic testing was not performed) that influence statin metabolism [6,7]. A key limitation in this case was the inability to perform anti-HMGCR antibody testing. A positive result would have favoured an IMNM phenotype, which typically requires immunosuppression and often does not resolve with statin withdrawal alone. Nevertheless, the patient’s favourable response to conservative management suggests that, if present, the immune-mediated component was not the dominant pathophysiological mechanism.

Vitamin D deficiency has an independent association with muscle weakness and myopathy, but its contribution to the severity of the presentation, including rhabdomyolysis, could not be quantified. Its supplementation as part of the management strategy was rational, though its specific attribution to the recovery could not be established.

Suggested mechanisms for neuropathy include possible alterations in nerve membrane function due to inhibition of cholesterol and coenzyme Q10, as well as other essential compounds derived from the mevalonate pathway [7,8]. Another postulated hypothesis is damage to the myelin sheath, leading to severe vacuolisation, though this has been shown in animal models [8]. The demyelinating pattern on NCV studies in this case is consistent with these proposed mechanisms.

This case has several practical clinical implications. First, statin history must be actively sought in any patient presenting with acute neuromuscular weakness, particularly when CK is significantly elevated, a finding inconsistent with GBS, and the diagnosis must be reviewed. Second, the mixed neuromuscular phenotype on NCV and EMG studies should not lead to diagnostic confusion. Third, statin discontinuation along with aggressive fluid resuscitation can achieve significant recovery even in severe presentation, and immunotherapy initiation can be prevented. Fourth, anti-HMGCR testing, while ideal, may not always be feasible in resource-limited settings; clinical reasoning and temporal relation to statin use remain central to diagnosis.

Another important part is to address the need for secondary cardiovascular prevention, as in our case. In such statin-intolerant patients, cautious reintroduction of statin therapy at a lower dose with close monitoring, alternate-day dosing, or the use of non-statin agents such as PCSK9 inhibitors may be considered.

## Conclusions

Statin-associated rhabdomyolysis can present with a clinical phenotype closely mimicking GBS with a distinctive mixed neuromuscular feature of concurrent demyelinating polyneuropathy on electrophysiological studies. This type of case, though clinically severe, may respond well to discontinuation of statins and supportive care without the need for immunotherapy. Clinicians should maintain a high index of suspicion for statin toxicity in older patients presenting with acute neuromuscular weakness, particularly in the setting of elevated CK, myoglobinuria, and recent statin initiation. Anti-HMGCR antibody testing remains an important investigation where resources permit. Future prospective studies are needed to characterise the mixed neuromuscular phenotype, define the role of immune-mediated mechanisms in neuropathic involvement, and establish standardised management guidelines for this rare but potentially reversible condition.
